# Multifactor Quality and Safety Analysis of Antimicrobial Drugs Sold by Online Pharmacies That Do Not Require a Prescription: Multiphase Observational, Content Analysis, and Product Evaluation Study

**DOI:** 10.2196/41834

**Published:** 2022-12-23

**Authors:** Tim Ken Mackey, Alan K Jarmusch, Qing Xu, Kunyang Sun, Aileen Lu, Shaden Aguirre, Jessica Lim, Simran Bhakta, Pieter C Dorrestein

**Affiliations:** 1 Global Health Program Department of Anthropology University of California, San Diego La Jolla, CA United States; 2 Global Health Policy and Data Institute San Diego, CA United States; 3 S-3 Research San Diego, CA United States; 4 Collaborative Mass Spectrometry Innovation Center Skaggs School of Pharmacy and Pharmaceutical Sciences University of California, San Diego La Jolla, CA United States; 5 Department of Healthcare Technology and Policy University of California, San Diego La Jolla, CA United States; 6 Department of Pharmacology University of California, San Diego La Jolla, CA United States; 7 Department of Pediatrics University of California, San Diego La Jolla, CA United States

**Keywords:** online pharmacy, antimicrobial resistance, drug safety, cyberpharmacies, public health, health website, online health, web surveillance, patient safety

## Abstract

**Background:**

Antimicrobial resistance is a significant global public health threat. However, the impact of sourcing potentially substandard and falsified antibiotics via the internet remains understudied, particularly in the context of access to and quality of common antibiotics. In response, this study conducted a multifactor quality and safety analysis of antibiotics sold and purchased via online pharmacies that did not require a prescription.

**Objective:**

The aim of this paper is to identify and characterize “no prescription” online pharmacies selling 5 common antibiotics and to assess the quality characteristics of samples through controlled test buys.

**Methods:**

We first used structured search queries associated with the international nonproprietary names of amoxicillin, azithromycin, amoxicillin and clavulanic acid, cephalexin, and ciprofloxacin to detect and characterize online pharmacies offering the sale of antibiotics without a prescription. Next, we conducted controlled test buys of antibiotics and conducted a visual inspection of packaging and contents for risk evaluation. Antibiotics were then analyzed using untargeted mass spectrometry (MS). MS data were used to determine if the claimed active pharmaceutical ingredient was present, and molecular networking was used to analyze MS data to detect drug analogs as well as possible adulterants and contaminants.

**Results:**

A total of 109 unique websites were identified that actively advertised direct-to-consumer sale of antibiotics without a prescription. From these websites, we successfully placed 27 orders, received 11 packages, and collected 1373 antibiotic product samples. Visual inspection resulted in all product packaging consisting of pill packs or blister packs and some concerning indicators of potential poor quality, falsification, and improper dispensing. Though all samples had the presence of stated active pharmaceutical ingredient, molecular networking revealed a number of drug analogs of unknown identity, as well as known impurities and contaminants.

**Conclusions:**

Our study used a multifactor approach, including web surveillance, test purchasing, and analytical chemistry, to assess risk factors associated with purchasing antibiotics online. Results provide evidence of possible safety risks, including substandard packaging and shipment, falsification of product information and markings, detection of undeclared chemicals, high variability of quality across samples, and payment for orders being defrauded. Beyond immediate patient safety risks, these falsified and substandard products could exacerbate the ongoing public health threat of antimicrobial resistance by circulating substandard product to patients.

## Introduction

The growing spread of antimicrobial resistance (AMR) is a global public health and security threat gaining increased attention from public health practitioners, clinicians, and policy makers alike [1]. The World Health Organization (WHO) estimates that US $1.2 trillion in additional health expenditure per annum is expected due to AMR by 2050 [2]. A recent systematic analysis estimated that AMR led to the direct cause of 1.27 million deaths in 2019 alone [3]. Hence, the future health, environmental, and economic costs of AMR have made it a priority global health issue that needs to be addressed [4].

Growth in AMR is driven by several factors, including misuse and overuse of prescription antibiotics that may not be rationally prescribed or subject to sufficient professional oversight, including nonprescription or over-the-counter dispensing, as well as potential illegal sourcing from nonauthorized channels [5-7]. This includes antibiotics sold via the internet, where documented direct-to-consumer sale can enable patients to choose their dosages, duration of treatment, and type of treatment, and enabling vendors to dispense the product without requiring a valid prescription [8-11]. These online pharmacies may also be conduits for patient exposure to substandard and falsified medicines, with anti-infective classes of medications widely reported as counterfeited and offered for sale online [6,12-15].

Due to the clear public health risks posed by online pharmacies engaged in questionable sourcing, previous studies have focused on conducting surveys of antibiotic purchasing behavior or examining characteristics of online sellers and the general availability of antibiotic products to better characterize the risk. For example, an early study on the topic published in 2009 identified 138 vendors selling antibiotics without a prescription and the sale of several antibiotic therapeutic classes by conducting “no prescription”–related keyword searches on Google and Yahoo search engines [16]. A more recent study published in 2017 reinforced these results by identifying 20 unique online pharmacies located in the United Kingdom offering the sale of antibiotics, with 45% not requiring a valid prescription [6]. Additionally, a 2020 study conducted a nationwide cross-section assessment of online and community pharmacies in China and found that 79% of online pharmacies did not require a valid prescription [7]. Though these studies provide important empirical evidence and reaffirm the use of the internet as an unregulated and potentially illegal point of access for antibiotics, no studies to our knowledge have used a combination of these methods to evaluate actual product safety and quality features of the products offered.

In response, this study expands on prior studies by first identifying and characterizing online pharmacies selling prescription antibiotics with a specific focus on common medications. We then conduct test purchases of antibiotics detected from “no prescription” online pharmacies and visually inspect packaging and products for possible safety concerns. Finally, the study conducts chemical analysis of antibiotic samples using mass spectrometry (MS) and molecular networking to assess quality characteristics and possible risk indicators.

## Methods

### Overview

**Figure 1:**
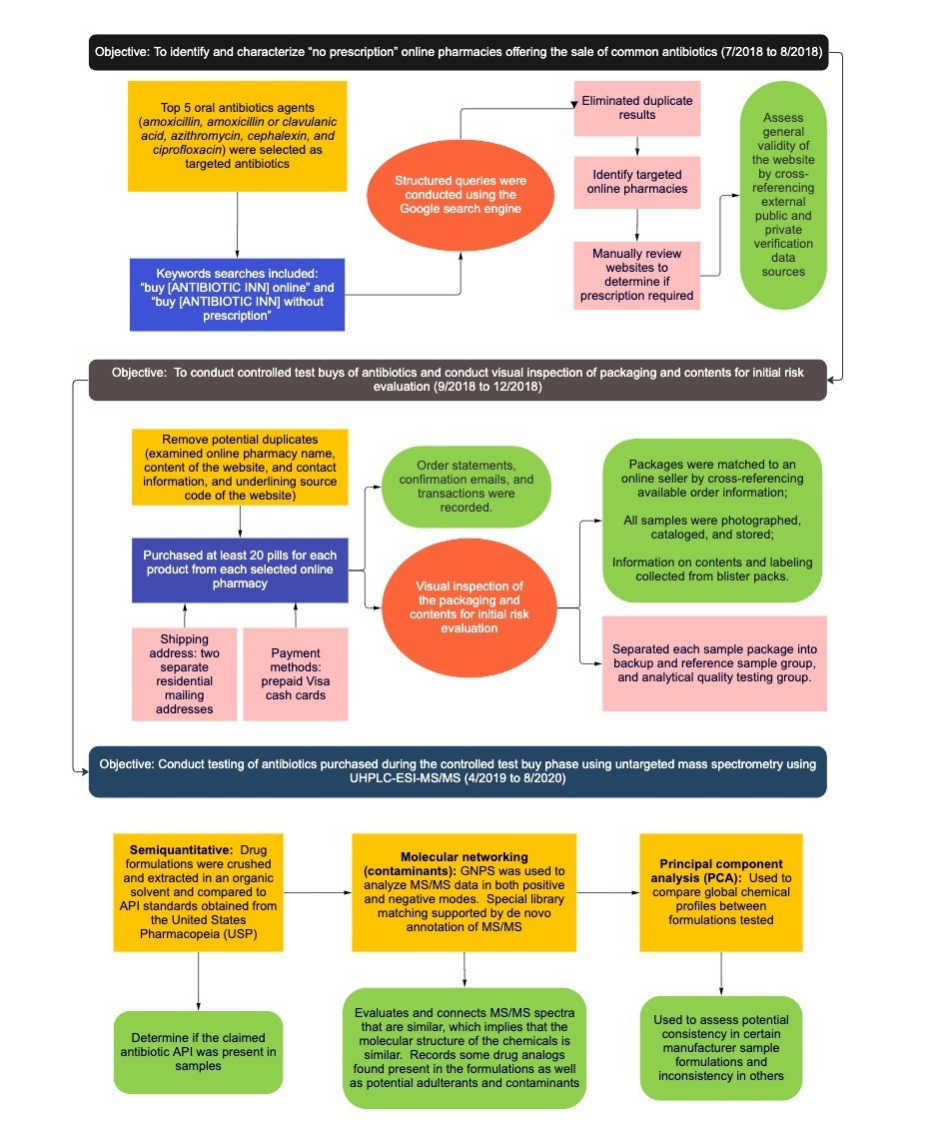
Study strategy and summary of methods. API: active pharmaceutical ingredient; GNPS: global natural product social molecular networking; MS: mass spectrometry; UHPLC-ESI-MS/MS: ultra–high performance liquid chromatography–electrospray ionization tandem mass spectrometry.

This multifactor risk and quality assessment was conducted in 3 phases (Figure 1). The first phase used structured search engine queries to identify and characterize “no prescription” online pharmacies offering the sale of common antibiotics. The second phase used websites identified in the first phase to conduct controlled test buys of antibiotics and conducted visual inspection of packaging and contents for risk evaluation. The third and final phase involved testing antibiotics purchased during the controlled test buy phase using untargeted mass spectrometry through ultra–high performance liquid chromatography–electrospray ionization tandem mass spectrometry. This study did not involve research on human participants. We describe each phase in detail below.

### Structured Web Searches

To identify websites and specific vendors advertising the sale of common antibiotics without a prescription, we selected the top 5 oral antibiotics agents prescribed to outpatient in the United States (amoxicillin, amoxicillin and clavulanic acid, azithromycin, cephalexin, and ciprofloxacin) according to data from the US Centers for Disease Control and Prevention (“targeted antibiotics”) as they constitute commonly prescribed antibiotics in the jurisdiction of focus of this study [17]. Structured queries were conducted using the Google search engine with cookie files removed and the incognito privacy setting selected in Chrome browser. Keyword searches included the following: “buy [ANTIBIOTIC] online” and “buy [ANTIBIOTIC] without prescription” repeated for all 5 targeted antibiotics using a protocol similar to other published studies (Multimedia Appendix 1) [6,18]. All URLs or hyperlinks populated in the first 10 pages of search results were included for analysis. Searches were conducted between April and May 2018.

After collating search engine results by cataloging returned URL or hyperlinks, we first eliminated all duplicate results and then conducted website content analysis to identify and classify websites that met the criteria for *online pharmacies*—websites that purport to operate as an internet pharmacy and include an e-commerce shopping cart to enable direct-to-consumer purchase (see Multimedia Appendix 1 for other website classifications assessed) [6,8,19,20]. URLs or websites that were classified as *online pharmacies* were then manually reviewed to determine if they required a valid prescription to place an order. This included assessing if the website claimed to be a “no prescription” online pharmacy and confirming whether a valid prescription was required prior to the ordering process, or if the website did not require a prescription or claimed to use a medical questionnaire in lieu of a prescription requirement.

We also assessed the general validity of the website or URL by cross-referencing external public and private verification data sources including LegitScript (a web-based service that monitors online pharmacies for compliance with applicable laws and regulations and classifies illegal and legitimate websites), the National Association of Boards of Pharmacy (NABP) Not Recommended List, and the European Union (EU) logo for online sale of medicines introduced by Directive 2011/62/EU (“EU Common Logo”). To further identify and characterize the potential location (both physical address and IP address) and owner of websites reviewed, we also cross-referenced data from the Internet Corporation for Assigned Names and Numbers, WHOIS lookup tool, to assess the registrar, registrant name, registrant country, registrant address, IP address, and IP Server for each URL. Content analysis of websites was conducted from May to June 2018.

### Controlled Test Buys and Visual Inspection

After completion of website classification, online pharmacies were evaluated for selection in our control test purchase phase using a study inclusion and exclusion criteria protocol (Multimedia Appendix 1). Beginning in July 2018, we began to conduct controlled purchases of targeted antibiotics from online pharmacies that purported to sell at least 1 of the 5 targeted antibiotics. To avoid placing orders from the same owner or group of multiple online pharmacies or affiliated websites, we examined the similarity of the online pharmacy name, content of the website, contact information, and underlining source code of the website (JavaScript) to remove potential duplicates. This generated a smaller sample of websites that comprised our final set of websites for our test purchase process.

Based on the final set of online pharmacies generated, all targeted antibiotics were advertised as sold by pack (eg, 15 pills/pack, 30 pills/pack), with the number of pills in a pack and the price varied. To generate enough samples for the phase 3 analytical quality testing, we purchased at least 20 pills for each product from each selected online pharmacy and set a minimum criterion for the dose of active ingredient (Multimedia Appendix 1). Purchases began in August 2018 and were made using prepaid Visa cash cards with shipment orders set to 2 separate residential mailing addresses on the West Coast of the United States. All order statements, confirmation emails, and transactions made to Visa cash card statements were recorded.

After receiving packages, we conducted visual inspection of packaging for initial risk evaluation. Packages were matched to an online seller by cross-referencing available order information, specific targeted antibiotics purchased, and cash card transaction record with all samples photographed, cataloged, and stored in a secure location. External packaging was then physically inspected for known product falsification risk characteristics and then opened for inspection and confirmation of antibiotics purchased based on information on blister packs (Table 1). We note that all packaging, regardless of country of origin, included English language markings on blister packages and labeling. We then separated each sample package into 2 groups, with the first group kept as a backup and reference sample and the second group used for purposes of analytical quality testing in phase 3 of the study.

**Table 1:** Risk characteristics of packaging and medicines’ visual inspection.

Risk characteristic		Description	Characteristics identified
**Packaging characteristics**			
	Type of package	Type of packaging used in shipments	Box Envelop
	Package damage	Inspect if there is any damage of the shipping package	Yes, package is damaged. No, package is in good condition.
	Postal shipping provider	Identification of shipping service or carrier used by the seller	India Post service Express Mail Service Prepaid Germany Postfach service Singapore Post United States Postal Service
	Shipping metadata	Return address and package tracking number	N/A^a^
**Item characteristics**			
	Types of drugs in each package	Detail of the content of each package	Quantity of drug in each package Identification and cataloging of drug formulations or names Identification of any unordered product or free samples in the package
	Prescription requirement warning	If “prescription required” warning is on the package (eg, blister pack or pouch) of drug	Yes, “prescription required” warning on the package No, no prescription required warning
	Type of package (factory packaging)	Types of medical packages for each type of drug	Blister packs Pouches
	Factory packaging damage	Inspect to determine if any damage to drugs shipped (how many)	Yes, (number of damaged drugs or pills) No, no damaged drugs

^a^N/A: not applicable.

### Analytical Quality Testing

Targeted antibiotics collected in the controlled test buy phase were then prepared for analytical testing and sent for analysis using untargeted MS through ultra–high performance liquid chromatography–electrospray ionization tandem mass spectrometry (Multimedia Appendix 1). Drug formulations were crushed and extracted in an organic solvent and compared to active pharmaceutical ingredient (API) standards obtained from the US Pharmacopeia. Semiquantitative results—defined as integrated MS signal over time, yielding peak areas that are reflective of amount but cannot be used to determine absolute physical quantity or concentration—were used to determine if the claimed antibiotic API was present in the samples. Molecular networking using the global natural product social molecular networking platform [21] was used to analyze MS/MS data in both positive and negative modes. Molecular networking evaluates and connects MS/MS spectra that are similar, which implies that the molecular structure of the chemicals is similar.

Data from targeted antibiotic samples were collected using an ultra-high performance liquid chromatograph (Vanquish, Thermo) coupled with an Orbitrap mass spectrometer (QExactive, Thermo). A processing method was created in Xcalibur (Thermo) to integrate the values (MS1 data used) of the APIs claimed to be in the drug formulations tested, including samples of sildenafil and tadalafil that were sent to study members unsolicited by vendors. By special library matching supported by de novo annotation of MS/MS, some drug analogs, as well as adulterants and contaminants, were found present in the formulations.

## Results

### Online Pharmacy Characteristics

We collected 109 unique URLs (from a total of 135 URLs), of which 98 (89.9%) were classified as an online pharmacy, and 21 (19.3%) online pharmacies registered more than one URL. The vast majority (n=85, 78.0%) of these websites were classified as either “rogue” or “unapproved” by Legitscript, and 62 (56.9%) were on the NABP Not Recommended List. Additionally, only 19 (17.4%) had the EU common logo (Table 2). Further, based on available WHOIS data, 20 (18.3%) used commercially available domain masking and enhanced privacy services to hide their location and ownership. Among websites with available location data, the top 5 registrant countries were the United States, Russia, Barbados, Canada, and the United Kingdom, though registered locations included a broader set of countries that covered North America, South America, Europe, and Asia. After applying our inclusion and exclusion criteria for selection for test purchasing, 27 online pharmacies were selected for controlled test buys.

**Table 2:** Online pharmacy verification summary (N=109).

Variable		Value, n (%)
**LegitScript**		
	Legitimate	2 (1.8)
	Certified	3 (2.8)
	No information	19 (17.4)
	Rogue	69 (63.3)
	Unapproved	16 (14.7)
**EU^a^ common logo**		
	Not verified	90 (82.6)
	Verified	19 (17.4)
**NABP^b^**		
	Verified	2 (1.8)
	Information is not available	26 (23.9)
	Not recommended list	62 (56.9)

^a^EU: European Union.

^b^NABP: National Association of Boards of Pharmacy.

### Controlled Buy Results and Packaging Analysis

Orders were placed with 27 online pharmacies and resulted in 1373 antibiotic product samples collected. This study defines product samples as a single pill or capsule that was collected from packaging sent to the research team, with packaging primarily consisting of blister packages with different numbers of pills or capsules. During the process of ordering, we received phone calls, as well as emails from vendors to confirm order details with phone calls not taken, but study team members responded to emails from vendors by simply confirming order details. Some online pharmacies included emails requesting the customer to verify transactions with the credit card–issuing bank, including emails with suspicious verification requests (eg, “If Support Bank can contact you for verification payment, please do not tell about buying medicines from XXX Pharmacy. You can tell them that you Paid for ‘FAMILY PHOTOS CONVERTED TO CD and FLASH DRIVE, or WEBSITE DESIGN ETC”).

Among all 27 orders, only 13 (48.1%) were successfully completed through the website’s online ordering process, which resulted in a confirmed transaction. However, 2 (7.4%) of these transactions resulted in fraud (eg, fraudulent purchases for other e-commerce transactions were made on Visa cash card information provided to online pharmacies), and we did not receive products from these vendors. From the remaining successfully completed transactions, 11 packages were received, and based on shipping labeling and records, 10 (91%) were identified as shipped from India and 1 (9%) from Singapore with all but 1 (9%) package shipped in a mailing envelope (ie, 1 package was delivered in a small box). In addition to the targeted antibiotics ordered, 2 (18.2%) packages included other unsolicited prescription drug products typically used or indicated for erectile dysfunction (eg, sidenafil citrate).

**Figure 2:**
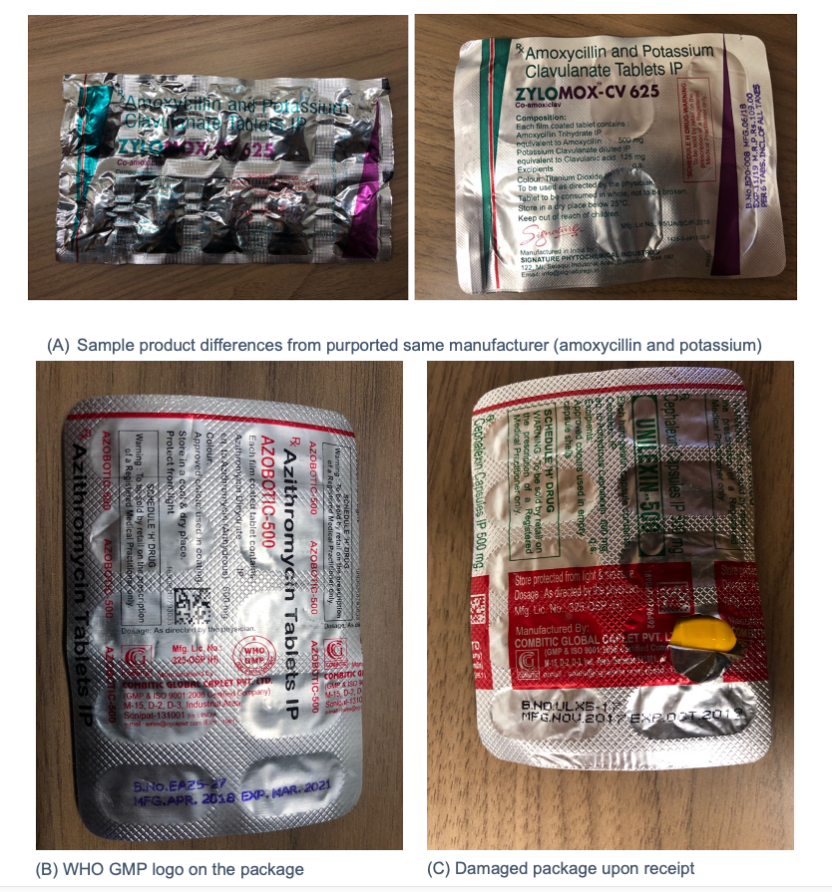
Image of risk characteristics identified from drug packages. GMP: Good Manufacturing Practices; WHO: World Health Organization.

Packages and samples were inspected for information on the name of the purported manufacturer, product warnings (eg, only dispensed with “Rx”), and any certification or logo indicating the authenticity of the product. Two envelopes had visible damage when received and contained damaged capsules when opened. Based on further packaging analysis, we found that among antibiotics, there was a high degree of variability in product characteristics (eg, color, shape, blister pack, etc), even among antibiotics purportedly from the same manufacturer. For example, in 2 packages received, both co-amoxicillin 625 mg samples are labeled as “Manufacturer X”; however, these 2 samples are packed and presented differently (Figure 2A). Some of the packages also included warning labels stating, “to be sold by retail on the prescription of a Registered Medical Practitioner only,” despite being purchased from sources that did not require a prescription. Finally, 2 blister packs also included a logo stating “WHO GMP certified company” (Figure 2B), though we were unable to confirm the validity of this certification or its origin (“WHO-GMP” certification could indicate certification from the Food and Drug Administration Maharashtra in India). Other products, as previously mentioned, arrived in damaged blister packs (Figure 2C).

### Analytical Testing of Samples

There was a total of 45 unique boxes or blister packs of drugs (from 12 manufacturers), from which 3 pills per package were analyzed through random selection, equating to 135 samples that underwent analytical testing. All targeted antibiotics purchased from internet pharmacies stated the name of the drug, stated on the label that it contained API, and, based on analytical testing, contained the stated API. However, certain samples had specific risk characteristics of interest, including chemicals that are presumed contaminants, drug-related compounds, and undeclared API.

Molecular networks via global natural product social molecular networking were used to explore differences in the untargeted MS data that might not have been listed in the formulations. Molecular networking and subsequent interpretation of potential risk factors revealed the presence of a feature annotated as octabenzone, a chemical used in sun-protection products in at least one sample from a manufacturer (sildenafil API listed) and 4 samples from another manufacturer (amoxicillin API listed; see Multimedia Appendix 1 for additional details). Dodecyl sulfate, a common surfactant, as well as tetradecyl sulfate were detected in 4 different manufacturers, which included cephalexin, ciprofloxacin, amoxicillin, and tadalafil as listed APIs, respectively. Among other chemicals observed in the molecular network, which we believe are contaminants, were flame retardants (triphenylphosphate), wetting and dispersing agents (tetramethyl-5-decyne-4,7-diol), and plasticizers. Regarding, drug-like chemicals, we observed a few chemicals related to the claimed API (connected in the molecular network to API) such as descladinose azithromycin as well as other unknown, unannotated chemicals. Finally, the sildenafil sample also contained dapoxetine (generally used as an API for the treatment of premature ejaculation), an undeclared API.

**Figure 3:**
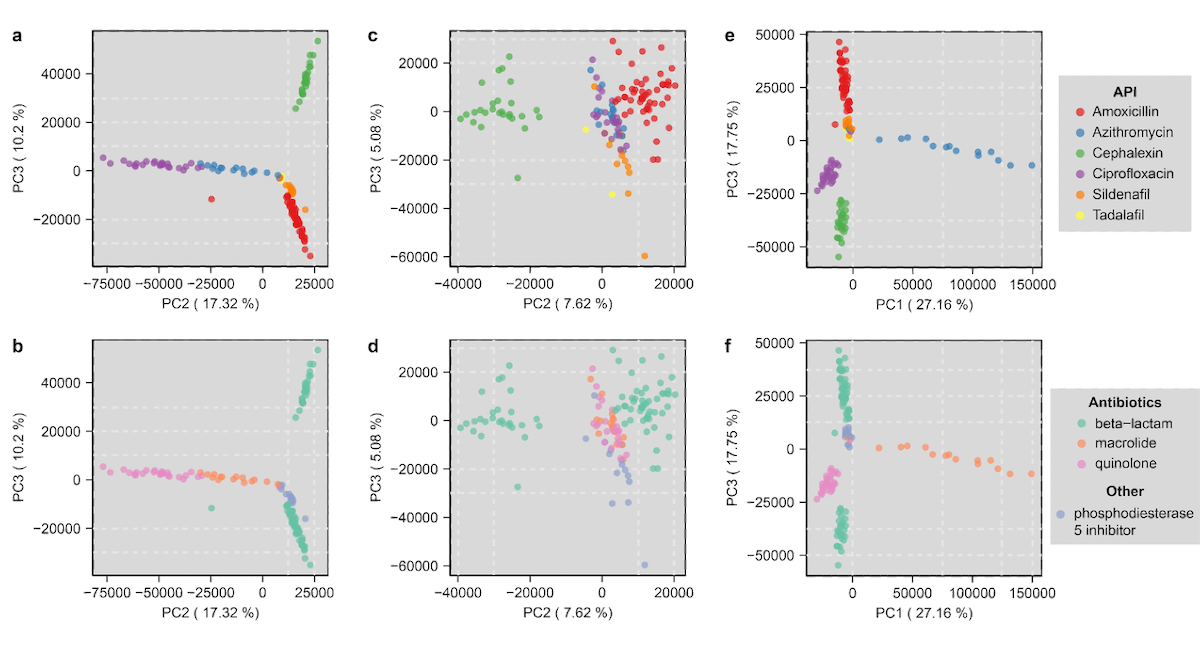
Untargeted mass spectrometric analysis of drugs formulations in positive and negative mode analyzed by principal component analysis (PCA). (a) PCA score plot of positive mode data, pareto scaled, displaying each sample as a point colored by active pharmaceutical ingredient (API) and (b) drug class; (c) PCA score plot of negative mode data, pareto scaled, colored by API and (d) drug class; (e) mid-level data fusion of positive and negative mode data displaying each sample as a point colored by API and (f) drug class.

Regarding variance across samples, untargeted MS analysis of the drug formulations using unsupervised multivariate statistics, specifically principal component analysis (PCA), was used to analyze the positive and negative mode data; in doing so, we compared the global chemical profiles between the formulations tested (Figure 3 and Multimedia Appendix 1). In the positive mode of PCA, separation of drug formulations was observed with clear grouping of samples based on the API observed in figure panels labelled principal component 2 (“PC2”) compared to principal component 3 (“PC3”), indicating consistency in certain manufacturer sample formulations and inconsistency in others (Figure 3A). PCA of negative mode data resulted in clear groups of amoxicillin and cephalexin, but less clear grouping of the other drug formulations, though overall, the chemical differences between samples were lesser in the negative mode compared to the positive ionization mode. Further analysis of the individual drug package and manufacturer compared to the overall separation observed by PCA by testing replicate samples indicated variances in chemical similarity of samples (Multimedia Appendix 1). Variance may result from differences in the quantitative amount of API (not fully evaluated in this study) or differences in the excipient and other chemicals present in the formulations tested.

## Discussion

### Principal Findings

Our study detected 109 unique websites actively advertising the sale of common antibiotics without a prescription, resulting in 27 online controlled test buy orders, 11 packages, and a total of 1373 antibiotic product samples that were evaluated using a combination of visual inspection, analytical chemistry, and molecular networking. In our sample of websites selling antibiotics, 57 masked their location or owner address with a privacy service, and of those ordered from, all the websites requested additional verification information in order to effectuate the processing of payment.

These characteristics potentially implicate risk factors for consumer and product safety associated with online drug purchasing and are generally considered characteristics of high-risk transactions that can result in identity theft or fraudulent transactions. Legitimate mail-order pharmacies do not exhibit these characteristics, and these risks are further reinforced by the lack of third-party validation for the majority of websites, as we found out after cross-referencing them with Legitscript, NABP, and MHRA verification. In fact, during the course of this study, prepaid cards used for test purchases were fraudulently charged (eg, fraudulent charges for food orders placed on prepaid cards without permission of the study team), and we were unable to recover these stolen funds.

Products shipped and received to us in the United States all originated from overseas, with almost all product samples shipped from India. As the focus of this study was from the context of a US patient or consumer, these antibiotics represent products outside of the controlled drug supply chain and have a higher risk of being adulterated, falsified, or otherwise harmful to human health. Almost all products were shipped in mailing envelopes, which did not have adequate protection to secure the product (some products appeared to be damaged in transit), and declared goods on labels often did not include “medicines” as a description, but instead were named as other unrelated consumer goods, likely in an attempt to evade customs inspection. These characteristics are clear warning signs of unauthorized and potentially falsified medications.

Finally, upon completion of analytical testing of samples using MS, all products were determined to have stated API, though this study did not quantitatively measure the percentage of API in each sample tested. We performed unsupervised multivariate statistics (eg, PCA) on the formulations, which indicated that particular manufacturers (as indicated on the packaging) were more precise in their formulation’s chemistry, whereas others displayed wider variability across replicate samples tested, possibly reflecting poor or inconsistent manufacturing practices or quality. The use of molecular networking also identified other impurities present in samples that evidence a further potential for adulteration, which may introduce unique patient safety harms.

Prior studies examining the quality of prescription drugs generally have findings along the following lines: they primarily focus on field-based sampling using different prevalence surveys and analytical techniques to test samples of antibiotics sold in physical establishments, primarily in low- to middle-income countries; they use packaging analysis or analytical techniques to test nonantibiotic drugs or dietary supplements (eg, erectile dysfunction drugs, growth hormone, diabetes drugs, stimulants, dietary supplements, etc) purchased from the internet; or they simply characterize different online sellers of antibiotics but do not purchase or test the products [13,22-31]. Our study builds on these prior studies by conducting a multifactor quality and safety analysis to generate new data points regarding the potential health and safety risks associated with the online sale of common antibiotics from no prescription providers that should be further confirmed through additional sampling and product testing. Importantly, many of the potential safety concerns identified are important in the context of broader public health and regulatory challenges aimed at addressing the global trade in substandard and falsified medicines, drug importation policy, ensuring supply chain integrity, and modernizing postmarket surveillance and pharmacovigilance approaches [32].

### Limitations

This study also has certain limitations given the methodology used. First, web-based search queries for marketing and the availability of drugs have certain limitations. Primarily, searches in this study were conducted at a limited time range, with an assessment of online availability that was also limited to the study period described. However, websites are created, modified, and taken down dynamically on the internet, hence limiting the generalizability of our results. Additionally, we cannot be certain if the stated manufacturer on the label or blister pack of samples was in fact the manufacturer of that product, as we did not contact manufacturers to confirm the medication lot number or authenticity. We also could not fully ascertain if visual inspection of packaging or sample quality was degraded, damaged, or underwent other spoilage due to shipping or storage issues prior to the product being received by the study team and independent of the online pharmacy, which could have impacted quality testing and external validity of results.

Finally, though this study focused on risks associated with importation of products from “no prescription” online pharmacies, the specific risk characteristics associated with antimicrobial drugs identified in this study may also be associated with different manufacturing standards or be indicative of a failure to manufacture drugs according to US standards from sources that originate outside of the United States. Future studies should incorporate additional control or comparison groups of online pharmacies (eg, foreign online pharmacies that require a valid prescription) to better understand factors associated with the identified risk characteristics.

### Conclusions

The use of infoveillance approaches, such as using structured web-based search queries, connecting results to “secret shopper” and online test buys, and evaluating sellers and products for risk characteristics, has the potential to address other online health challenges that may implicate illegal actors, such as the illegal sale of other prescription drugs, controlled substances, and illicit drugs, and even COVID-19–fraudulent products [11,33-35]. Critical to these efforts will be consensus building and the development of internationally agreed-upon standards and methodologies for initial risk evaluation when purchasing drugs online [36]. Comprehensive and specific risk assessments tailored for online sellers and even different drug classes can be developed based upon existing instruments, such as the European Directorate for the Quality of Medicines and the Asia Pacific Economic Cooperation Supply Chain Security toolkit for internet sales; weighted criteria for counterfeiting risk assessment, as suggested by Vida et al [36], can also be beneficial. The results from this study can also form the basis for future risk-based multimodal surveillance approaches and product quality assessment methodologies that can be scaled to larger data collection to establish more generalizable findings. For example, future studies should consider testing for the WHO Model Essential Medicine List ”WATCH” and “RESERVE” antibiotics given their potential for higher microbial resistance and public health impact, should they be counterfeited.

## Appendix

Multimedia Appendix 1 Supplementary file.

## Abbreviations

AMR: antimicrobial resistance
API: active pharmaceutical ingredient
EU: European Union
MS: mass spectrometry
NABP: National Association of Boards of Pharmacy
PCA: principal component analysis
WHO: World Health Organization

## Data Availability

Study data are available upon reasonable request to the authors. Mass spectrometry data are available on the internet [37].
