# Supplementary material for: Multifactor Quality and Safety Analysis of Antimicrobial Drugs Sold by Online Pharmacies That Do Not Require a Prescription: Multiphase Observational, Content Analysis, and Product Evaluation Study
Source: JMIR Public Health Surveill. 2022 Dec 23;8(12):e41834. doi: 10.2196/41834 (PMC9823576; doi:10.2196/41834)
Supplement: Multimedia Appendix 1 [file publichealth_v8i12e41834_app1.pdf]

Multimedia Appendix

Methods

Structured Web Searches: For all Targeted Antibiotic, we conducted two independent structured search queries using different study keywords for Targeted Antibiotics. For example, when we search for Amoxicillin, we conduct the first search by using the term "buy Amoxicillin online", and the second search by using term "buy Amoxicillin without prescription". We limited our final sample of websites reviewed to English language websites. For each search, by changing keywords and different antibiotics' names, results from the first 10 pages of websites were reviewed. Results containing news, academic papers, and websites not available were excluded.

Website classification: After collecting search engine results by cataloging returned URLs/hyperlinks, we first eliminated all duplicate results and then used website content analysis approaches to identify and classify websites into different types of seller categories including: (a) *online pharmacies* – websites that purport to operate as an Internet pharmacy and include an e-commerce shopping cart for direct-to-consumer purchase and shipment; (b) *individual drug selling offers* – a website or Internet-based post that includes contact information to order a drug directly from a purported seller; and (c) *marketing affiliate site* – websites that do not directly sell drugs to consumers, but instead refers online users to other websites (including online pharmacies) and generally earn commission through website referral links.

Controlled Test Buys and Packaging Inspection: Inclusion and exclusion criteria for test buy selection are as follows: We excluded 9 websites where the URLs were no longer available when re-evaluating for test purchasing. We also removed 23 online pharmacies that explicitly stated that they did not provide services to the United States, and 3 that required a valid prescription in order to place an order. Further, we excluded 10 online pharmacies that did not accept visa card as a payment method and 26 websites that we suspected as duplicate vendors based on same vendor contact information or website java script code (See **Multimedia Appendix Figure 1** for examples)

Multimedia Appendix Figure 1: Examples of Duplicate Websites

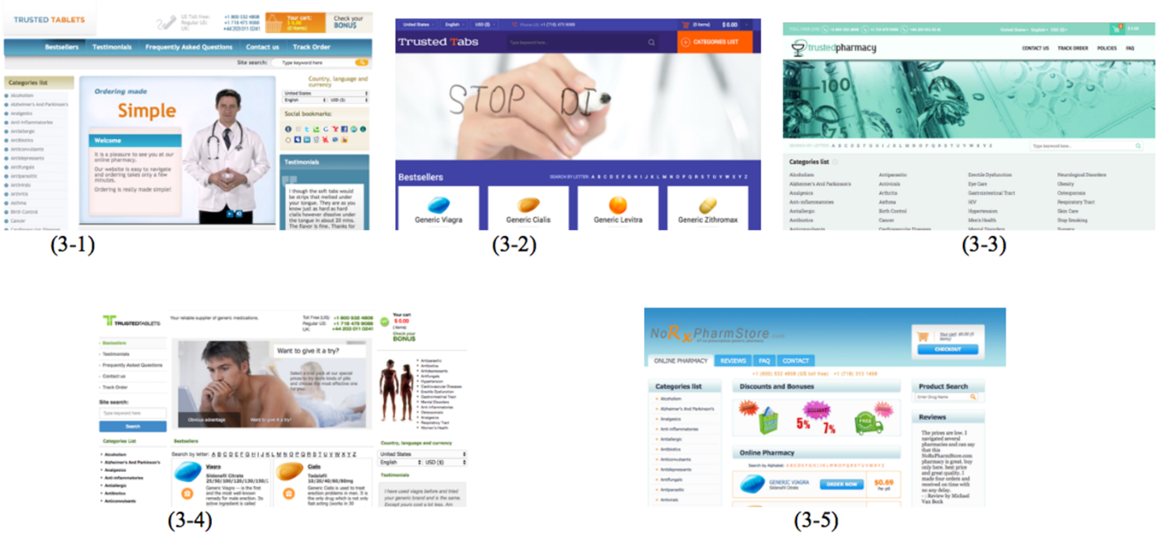

Five websites categorized as online pharmacy in our research: TRUSTED TABLETS (3-1), Trusted Tabs (3-2), trustedpharmacy (3-3), TRUSTEDTABLETS (3-4) and NoRxPharmStore.com (3-5). These 5 online pharmacies have different URLs domain name and format, but have a same phone number "+1(718)475 9088" as the contact number.

Geographic locations:

Multimedia Appendix Figure 2: Website Registrant Locations

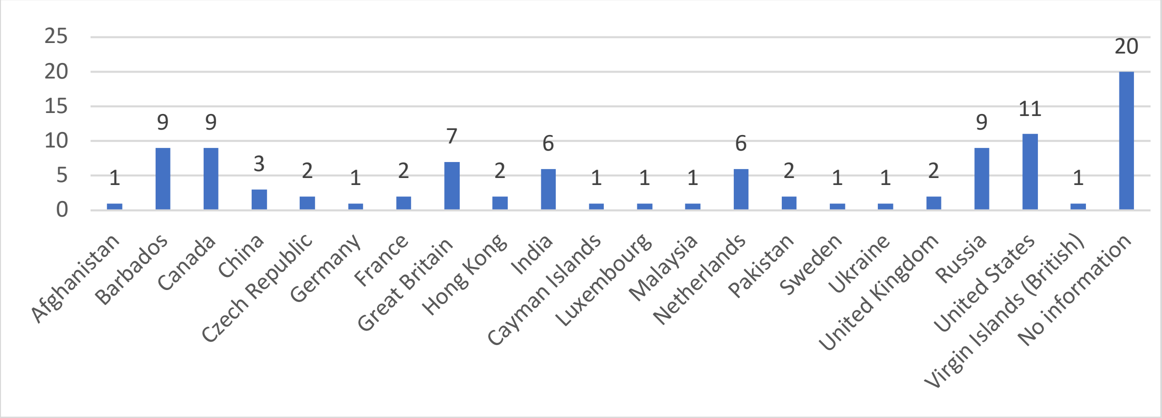

Product requirement: Minimum dosage criteria for active ingredient of Targeted Antibiotics: Amoxicillin- 500mg, Azithromycin- 500mg, Amoxicillin/clavulanic acid 625mg (500mg+125mg), Cephalexin- 500mg and Ciprofloxacin-500mg.

Sample Management: In order to manage study samples, we numbered each type of antibiotic from each package with a number combined with the package number and sample number. The received packages were numbered from “Package 1” to “Package 11”, and numbered Amoxicillin as sample No.1, Azithromycin as sample No.2, Cephalexin as sample No.3, Amoxicillin/clavulanic acid as sample No.4, Ciprofloxacin as sample No.5, unsolicited Nizagara as NO.6, unsolicited Vidalista 20 as No.7, and unsolicited Sildenafil citrate sildito as No.8. See **Multimedia Appendix Tables 1 and 2**.

Multimedia Appendix Table 1: Sample Testing Group

**Package Number**	*Convention X- [package number] XX [sample number]*							
	**No (01) Amoxicillin**	**NO (02) Azithromycin**	**NO(03) Cephalexin**	**NO (04) Co-Amoxicillin (Augmentin)**	**NO (05) Ciprofloxacin**	**NO (06) Free Nizagara**	**NO (07) Free Vidalista 20**	**NO (08) SidenafilCitratel Silditop**
1	500 mg*20	500mg*20	500mg*20	625 mg*18	500mg*40	100mg*4		
2			500mg*20	625 mg*20	500mg*20			
3	500 mg*20		500mg*20	625 mg*18	500mg*40	100mg*4		
4	500 mg*20	500mg*20	500mg*20	625 mg*18	500mg*40	100mg*4		
5	500mg*15		500mg*20	625mg*18	500mg*40			100mg*10
6	500mg*20	500mg*20	500mg*20	625mg*18	500mg*20			
7	500mg*15		500mg*20	625mg*20	500mg*40 (Ceflox)		20mg*10	
8		500mg*15						
9					500mg*20			
10	500mg*15	500mg*18	500mg*20	625mg*20	500mg*20			
11	500mg*20		500mg*20	625mg*20				

Multimedia Appendix Table 2: Sample Backup Group

Package Number	*Convention X- [package number] XX [sample number]*							
	**No (01) Amoxicillin**	**NO (02) Azithromycin**	**NO(03) Cephalexin**	**NO (04) Co-Amoxicillin (Augmentin)**	**NO (05) Ciprofloxacin**	**NO (06) Free Nizagara**	**NO (07) Free Vidalista 20**	**NO (08) SidenafilCitratel Silditop**
1	500 mg*10	500mg*10	500mg*10	625 mg*12	500mg*20	X		
2			500mg*10	625 mg*10	500mg*10			
3	500 mg*10		500mg*10	625 mg*12	500mg*20	X		
4	500 mg*10	500mg*10	500mg*10	625 mg*12	500mg*20	X		
5	500mg*15		500mg*10	625mg*12	500mg*20			100mg*7
6	500mg*20	500mg*20	500mg*20	625mg*18	500mg*20			
7	500mg*15		500mg*10	625mg*10	500mg*20 (Ceflox)		20mg*6	
8		500mg*9						
9					500mg*10			
10	500mg*15	500mg*12	500mg*10	625mg*10	500mg*10			
11	500mg*10		500mg*10	625mg*10				

Analytical Chemical Testing Details: Data from Targeted Antibiotic samples were collected using an ultra-high performance liquid chromatograph (Vanquish, Thermo) coupled with an Orbitrap mass spectrometer (QExactive, Thermo). Chromatographic separation was carried out on the analytical C18 column (with C18 guard cartridge) maintained at 40°C during separation. 5.0 µL of extract was injected per sample. Heated electrospray ionization (HESI) was performed in positive and negative ion mode (subsequent injections). Negative and positive mode data were collected using the same data-dependent acquisition parameters. MS1 scans were collected at 30,000 resolution from m/z 150 to 1500 performed (~7 Hz) with a maximum injection time of 100 ms, 1 microscan, and an automatic gain control target of 1x106. The top 3 most abundant precursor ions in the MS1 scan were selected for fragmentation with an m/z isolation width of 1.5 and subsequently fragmented with stepped normalized collision energy of 20, 30, and 40. The MS2 data was collected at 17,500 resolution with a maximum injection time of 100 ms, 1 microscan, and an automatic gain control target of 5x105.

For GNPS analysis, the positive mode feature-based molecular networking job can be accessed (https://gnps.ucsd.edu/ProteoSAFe/status.jsp?task=0834479d2e2c487994aa6f68253a209d). The negative mode feature-based molecular networking job can be accessed (https://gnps.ucsd.edu/ProteoSAFe/status.jsp?task=a40b5a63ef4d4e16830a7293f5a3181f). The data are freely available at MassIVE (massive.ucsd.edu) under accession number MSV000083605. The code and files used to produce the figures are available freely on GitHub (https://github.com/alan-jarmusch/WebDrugs).

Results

Controlled Test Buy Characteristics and Contents: A total of 11 packages were received and based on shipping labeling and records (see **Multimedia Appendix Table 4** for details). Based on the information from the package shipping tags, we found that 6 of them were shipped by India Post service, 2 of them were shipped by EMS service, 1 Prepaid Germany Postfach service, 1 Singapore Post/USPS and 1 ASLPSMNR. Additional unsolicited erectile dysfunction drugs included 4 pills of 100mg Nizagara in Package 1, Package 3 and Package 4; 10 pills of 100g Sidenafil Citrate Silditop in Package5, and 10 Pills of 20mg Vidalista in Package 7. Stated Manufacturers associated with test buy products were cross referenced with existing publicly available data from the World Health Organization Prequalification of Medical Products database to assess if any were listed as authorized pre-qualification manufacturers. Of these

manufacturers only one manufacturer of Ciprofloxacin (hydrochloride) was listed in the WHO Approved International Nonproprietary Name list as a manufacturer at the time of this study.

Multimedia Appendix Table 3: Online Pharmacy Order Details

**Website Number**	Amoxicillin 500mg (tablets)	Azithromycin 500mg (tablets)	Amoxicillin/ Clavulanic Acid 625 mg/635mg (tablets)	Cephalexin 500mg (tablets)	Ciprofloxacin 500mg (tablets)
**A**	not found	not found	Available (625mg,30tabels)	Available (30tablets)	Available (30tablets)
**B**	Available (180)	not found	not found	Available (90)	Available (90)
**C**	Available (20)	not found	Available (20)	Available (30)	Available (20)
**D**	Available (30)	not found	Available (635mg* 30)	Available (30)	Available (60)
**E**	Available (30)	not found	Available (625mg*30)	Available (30)	Available (60)
**F**	Available (30)	not found	Available (625mg*30)	Available (30)	Available (30)
**G**	Available (30)	not found	Available (625mg*30)	Available (30)	Available (30)
**H**	Available (30)	Available (30)	Available (625mg*30)	Available (30)	Available (30)
**I**	Available (30)	Available (30)	Available (635mg*30)	Available (30)	Available (30)
**J**	Available (30)	Available (30)	Available (625mg*30)	Available (30)	Available (60)
**K**	Available (30)	Available (30)	Available (635mg*20)	$63.99(30)	$43.99(30)
**L**	Available (30)	not found	Available (625mg*30)	$50.22(30)	$38.47(20)
**M**	Available (30)	Available (30)	Available (625mg*30)	Available (30)	Available (20)
**N**	Available (30)	Available (30)	Available (635mg*30)	Available (30)	Available (30)
**O**	Available (30)	Available (30)	Available (624mg*30)	Available (30)	Available (30)
**P**	Available (30)	Available (9+15)	Available (625mg*30)	Available (30)	Available (30)
**Q**	Available (30)	Available (24)	Available (30)	Available (30)	Available (30)
**R**	Available (30)	Available (30)	Available (635mg* 30)	Available (30)	Available (60)
**S**	Available (30)	Available (30)	Available (625mg*30)	Available (30)	Available (30)
**T**	Available (30)	Available (30)	Available (625mg*30)	Available (30)	Available (30)
**U**	Available (30)	Available (30)	Available (625*30)	Available (30)	Available (60)
**V**	Not found	Available (250mg*24)	Available (625mg*30)	Available (50)	Available (250mg*24)
**W**	Available (30)	Available (30)	Available (30)	Available (30)	Available 30)
**X**	Available (20tablets)	Available (30)	Available (20)	Available (30tablets)	Available (20)

**Y**	Available (30)	Available (9+15)	Available (30)	Available (30)	Available (30)
**Z**	not found	Available (20)	Available (625mg*20)	Available (20)	Available (20)
**AA**	Available (30)	Available(30)	Available (625*30)	Available (30)	Available (60)

Analytical Testing:

Untargeted MS analysis of the drug formulations resulted in the detection of 1,898 MS features in the positive ionization mode and 2,125 MS features in the negative ionization mode. Unsupervised multivariate statistics, specifically principal component analysis (PCA), was used to analyze the positive and negative mode data and in doing so compare the global chemical profiles between formulations. In positive mode of PCA, separation of drug formulations was observed with clear grouping of samples based on API observed in figure panels PC2 versus PC3 (see **Figure 3** in main manuscript text). Further, separation of drug classes was noted, for example, the beta-lactam antibiotics were separate from the macrolide and quinolone antibiotics along the PC2 axis. The MS features contributing to the observed separation are displayed in Figure **Multimedia Appendix Figure 3-S1a** including the APIs which were confirmed by MS/MS spectral matching via the GNPS platform. For example, the separation of ciprofloxacin samples in Figure S1a in the negative direction along the PC2 axes is due to, in part, features 499 and 431 which were annotated as ciprofloxacin (**Figure 3-S1a**).

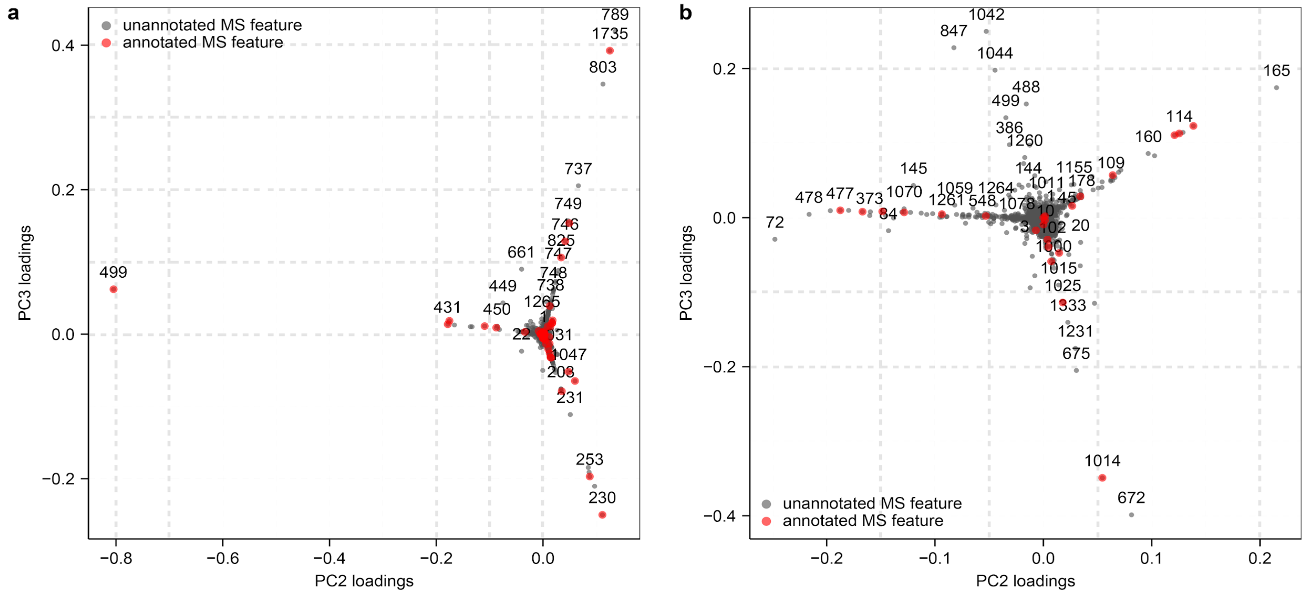

Figure S1. PCA Loading Plot for Positive and Negative Mode Data. (a) PCA loading plot for positive mode data. Unannotated MS features are plotted as grey points and annotated, via MS/MS spectral matching in GNPS, are overlaid as red points. (b) PCA loading plot for negative mode data with unannotated MS feature (grey points) and annotated features (red points).

PCA of negative mode data resulted in clear groups of amoxicillin and cephalexin, but less clear grouping of the other drug formulations (**Figure 3c in** main manuscript). The MS features contributing to the separation are displayed in **Figure 3-S1b**. Overall, the chemical differences between samples were lesser in the negative mode compared to the positive ionization mode as

supported by the variance contributed by the PCs (**Figure S2a-b**). Mid-level data fusion, *i.e.* combination of the first 8 PCs of positive and negative mode and reanalysis by PCA, was performed and resulted in clear separation of drug formulations by API and drug class (**Figure 1e-1f** in main text**)**. A mid-level data fusion strategy combines the information in the positive and negative mode and results in more of the variance explained by the first 8 PCs (**Figure 3-S2c**).

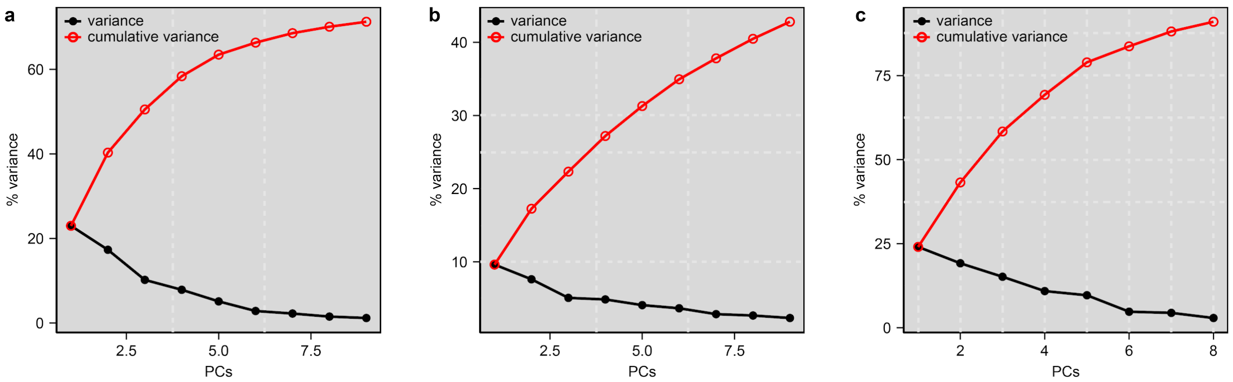

Figure S2. Variance and Cumulative Variance from PCA. (a) Positive mode data variance (closed, black points) and cumulative variance (open, red points). (b) Negative mode data variance (closed, black points) and cumulative variance (open, red points). (c) Mid-level data fusion variance (closed, black points) and cumulative variance (open, red points).

While the drug formulations were found to group based on the API, the individual drug package and manufacturer were evaluated compared to the overall separation observed by PCA on positive and negative mode data. Replicate samples, 3 samples from each package, were analyzed. The location of those samples in the PCA score plots are displayed for positive mode, negative mode, and mid-level data fusion (**Figure 3-S3-5**, respectively). Within the positive mode data, the grouping of replicate drug formulations, implying chemical similarity, varied. For example in amoxicillin samples (**Figure 3-S3**), the formulation of manufacturer 8 from package 1_1 displayed greater variance (lesser grouping) than the same manufacturer's package 3_1. The grouping in negative mode (**Figure 3-S4**) appeared to be more similar overall compared to the positive mode data. And the data fusion indicated variances in grouping, likely the contribution of the positive mode data. Further, it should be noted that the overall grouping of formulations varied as well as the location of each formulation tested. For example, the grouping of ciprofloxacin formulations in the data fusion (**Figure 3-S5**) results was more closely grouped than that of azithromycin. And while certain formulations spanned the larger azithromycin group (*e.g.* manufacturer 2, package 1_2) others were closely grouped at either end of the grouping (*e.g.* manufacturer 1, package 10_2 versus manufacturer 6, package 8_2). This may result from differences in quantitative amount of API (not evaluated here) or differences in the excipient and other chemicals present in the formulations tested.

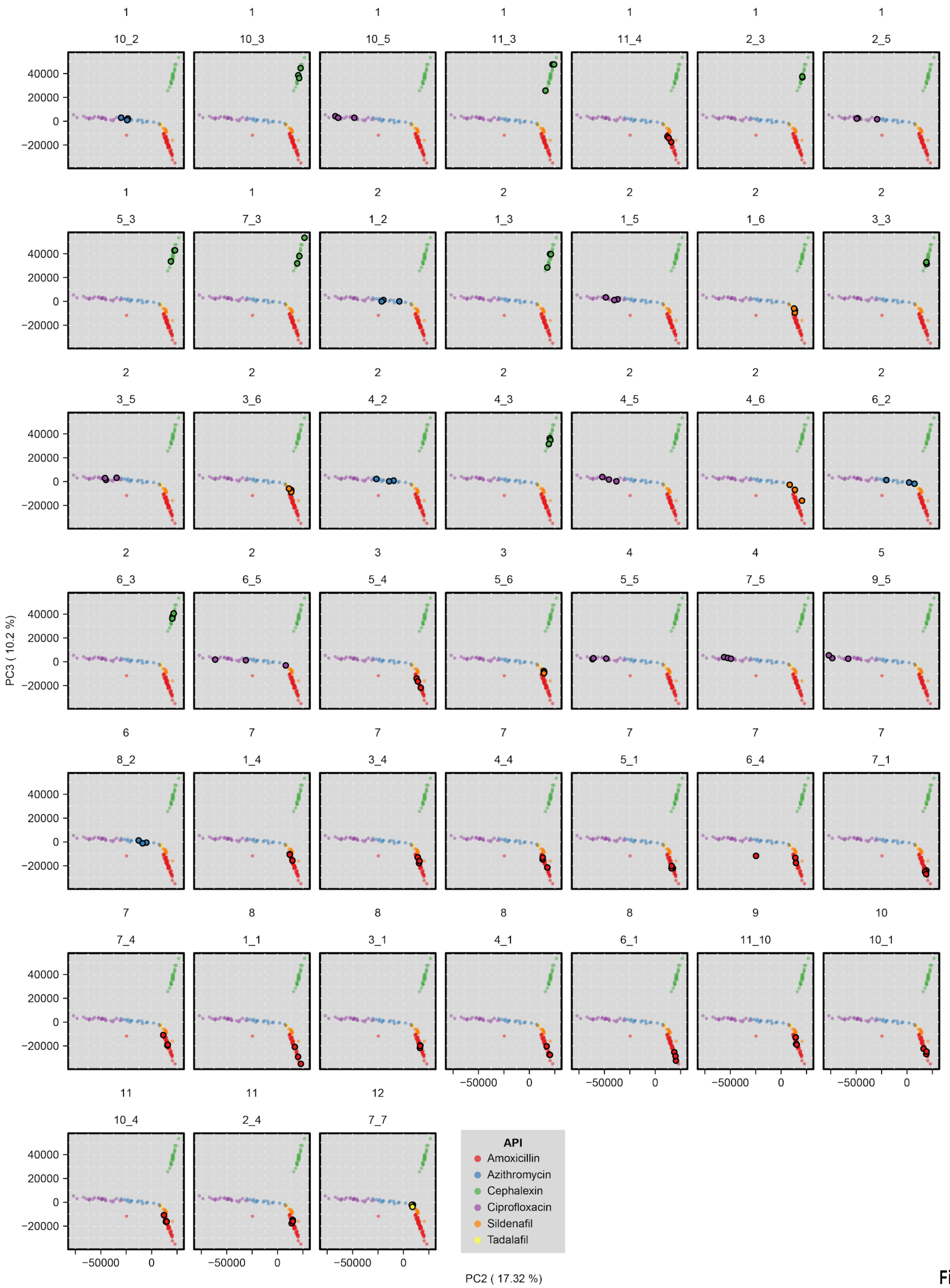

Figure S3. Untargeted Mass Spectrometric Analysis of Drugs by Manufacturer in Positive Mode Analyzed by PCA. PCA score plot colored by API faceted by the packaging number and manufacturer. Samples corresponding to the packaging number and manufacturer in each facet are overlaid, points are outlined in black.

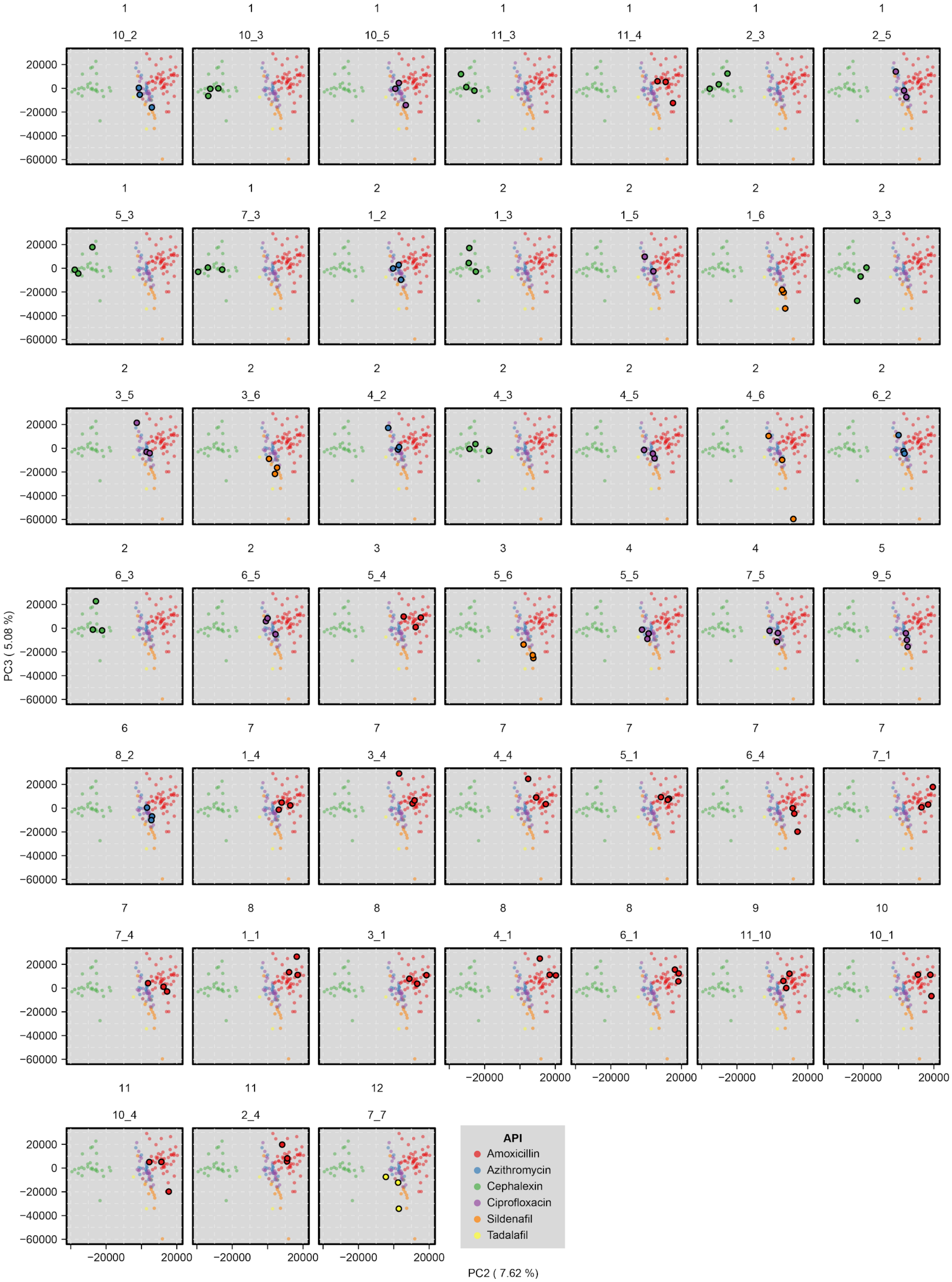

Figure S4. Untargeted Mass Spectrometric Analysis of Drugs by Manufacturer in Negative Mode Analyzed by PCA. PCA score plot colored by API faceted by the packaging number and manufacturer. Samples corresponding to the packaging number and manufacturer in each facet are overlaid, points are outlined in black.

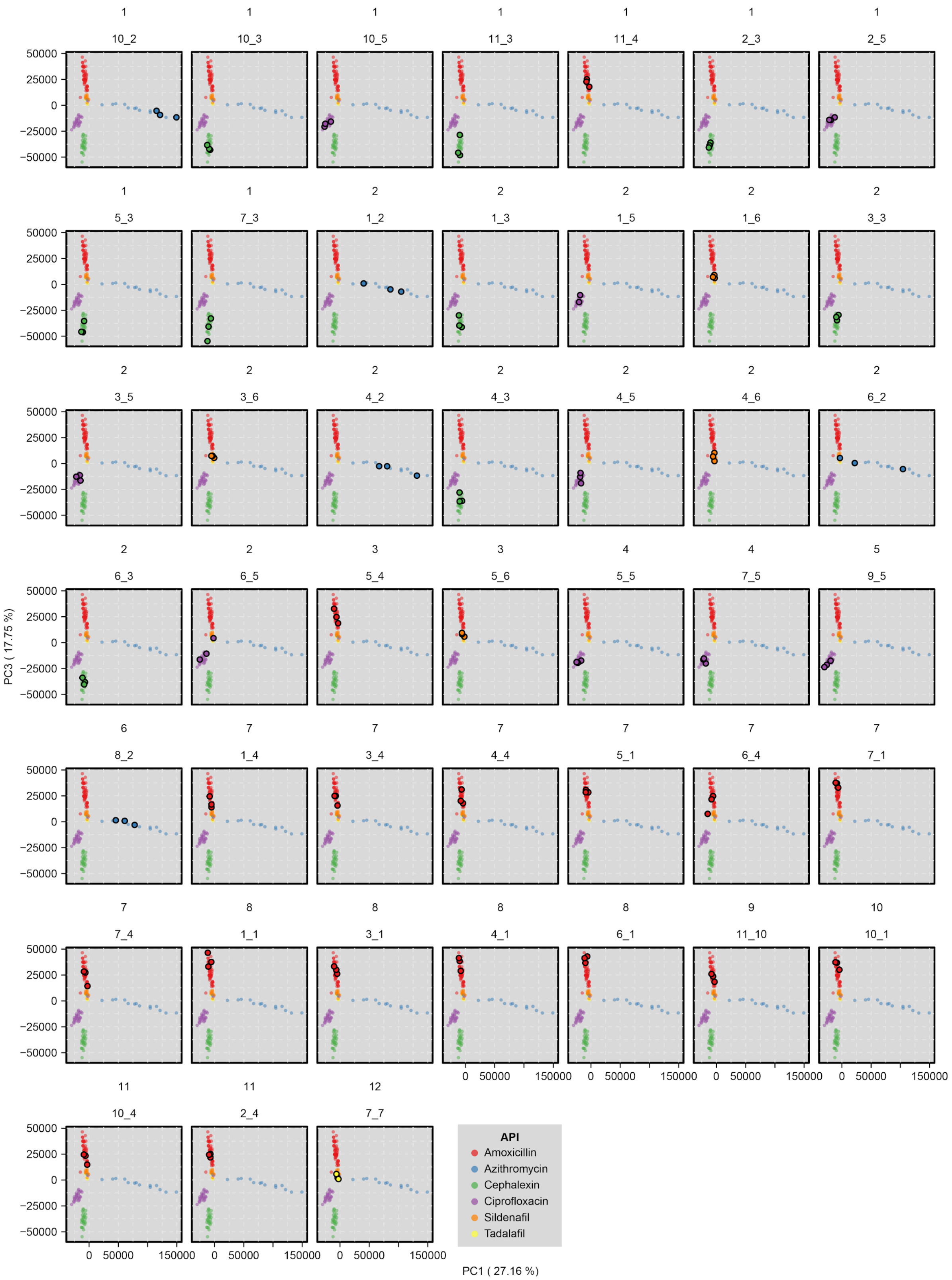

Figure S5. Untargeted Mass Spectrometric Analysis of Drugs by Manufacturer in Mid-level Fused Positive and Negative Mode Analyzed by PCA. PCA score plot colored by API faceted by the packaging number and manufacturer. Samples corresponding to the packaging number and manufacturer in each facet are overlaid, points are outlined in black.

A more detailed chemical exploration of the positive and negative mode data, specifically the MS/MS data that were acquired by data-dependent acquisition, was performed via molecular networking in GNPS. The molecular networks for the positive and negative mode are displayed in **Figure 3-S6** with annotated chemicals, level 2 based on MS/MS spectral library matching, are highlighted in red. The molecular networks were used to explore differences in chemicals that might not have been listed in the formulations. While the vast majority of nodes (proxy for MS/MS spectra) were unannotated, those which were annotated were found to display formulation specific distributions. For example, a spectral match to octabenzone, a chemical commonly used in sun protection products, was found in manufacturer 4, formulation 5_5 as well as a number of manufacturer 7's formulations (**Figure 3-S7a**). Similarly, 2,4,7,9-Tetramethyl-5-decyne-4,7-diol, a wetting, anti-foaming, and dispersing agent, was annotated from the positive mode data and was detected in a number of formulations and manufacturers (**Figure 3-S7b**).

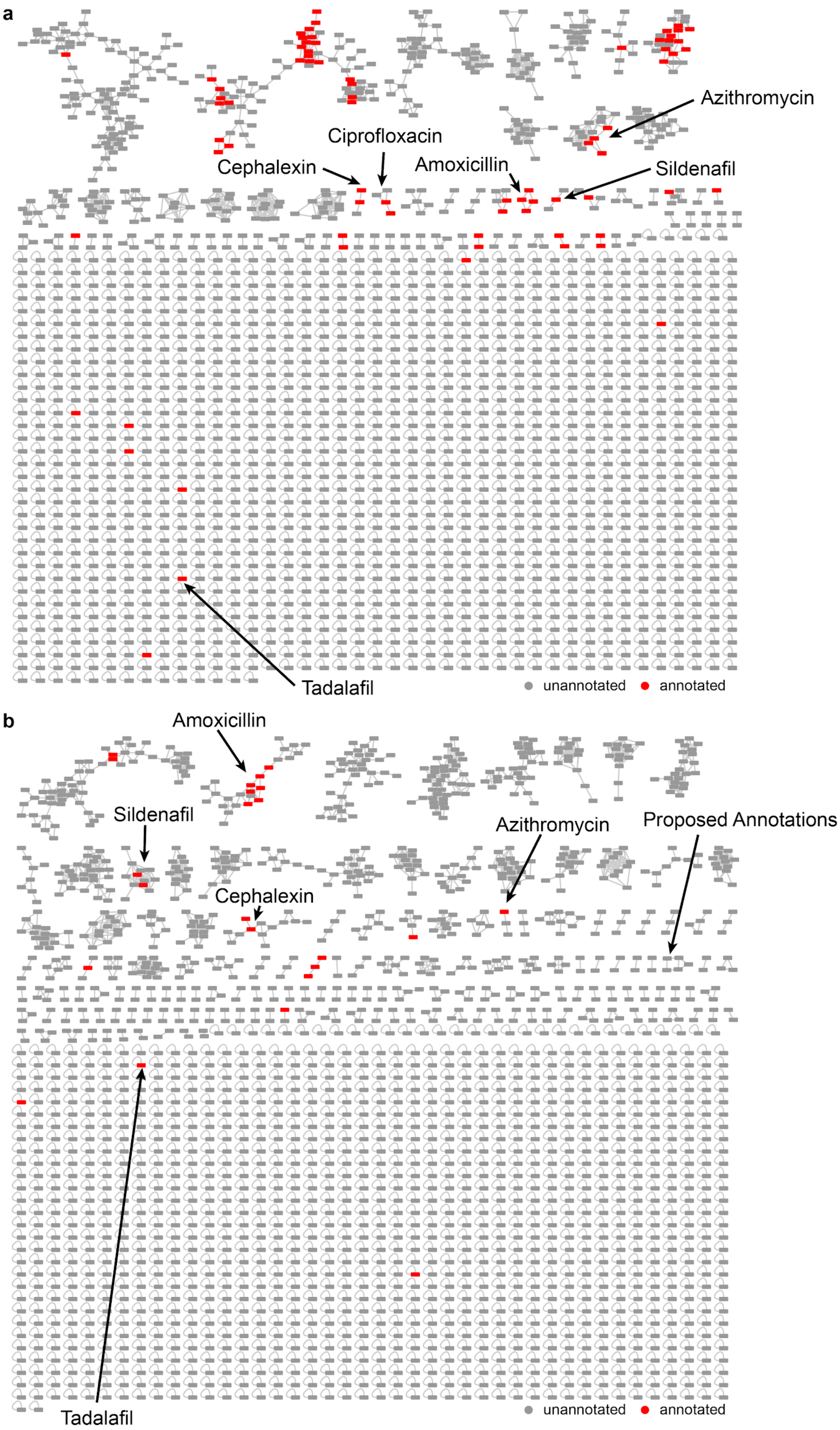
a
Azithromycin
Ciprofloxacin
Cephalexin
Amoxicillin
Sildenafil
Tadalafil
unannotated
annotated
b
Amoxicillin
Sildenafil
Azithromycin
Proposed Annotations
Cephalexin
Tadalafil
unannotated
annotated

Figure S6. Molecular Networking via GNPS of Positive and Negative Mode. (a) Molecular network resulting from the analysis of MS/MS data in the positive mode. (b) Molecular network of negative mode MS/MS data. Annotated nodes, based on MS/MS spectral library matching, in the molecular network are red and unannotated nodes are colored grey. The proposed annotations indicated in (b) correspond to **Figure S8**.

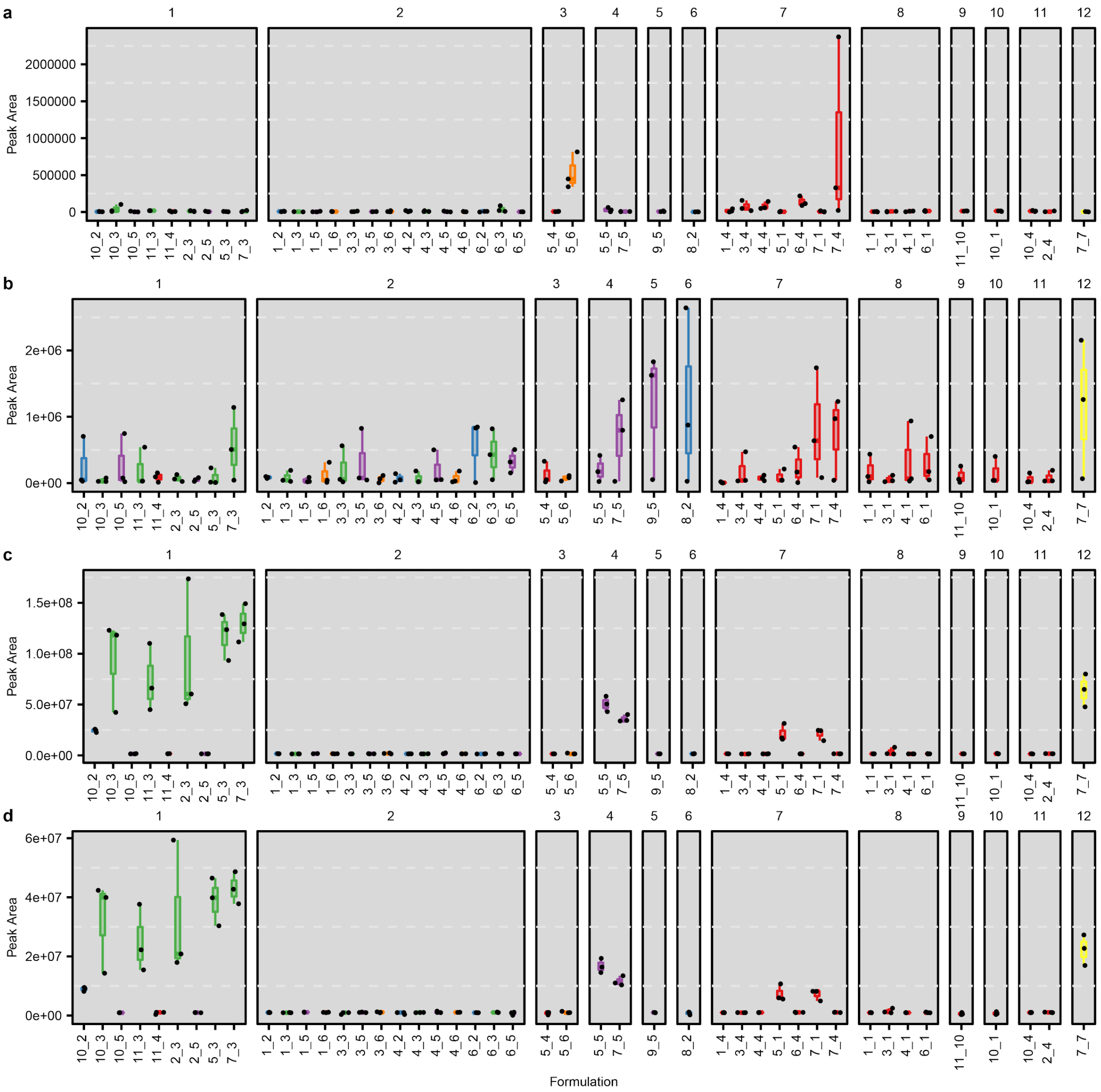

Figure S7. Box and Whisker Plots for Drug Formulations Organized by Manufacturer. (a) Annotation of octabenzone (feature # 1230, *m/z* 327.2008, and retention time of 2.13 min) in the positive ionization mode. (b) Annotation of 2,4,7,9-Tetramethyl-5-decyne-4,7-diol (feature #1452, *m/z* 209.1902, and retention time of 4.53 min) in the positive mode. (c) Proposed annotation of dodecyl sulfate in the negative mode (feature #72, *m/z* 265.1487, and retention time of 4.79 min). (d) Proposed annotation of tetradecyl sulfate (feature #84, *m/z* 293.1803, and retention time of 5.35 min) in the negative mode. Faceted by the manufacturer. Boxes indicate the 25%, 50%, and 75% quantiles and the whiskers extend ±1.5 times the interquartile range. Colors reflect claimed API: amoxicillin (red), azithromycin (blue), cephalexin (green), ciprofloxacin (purple), sildenafil (orange), and tadalafil (yellow). Annotations are level 2 according to the Metabolomics Standards Initiative (Sumner et al., 2007) unless otherwise specified.

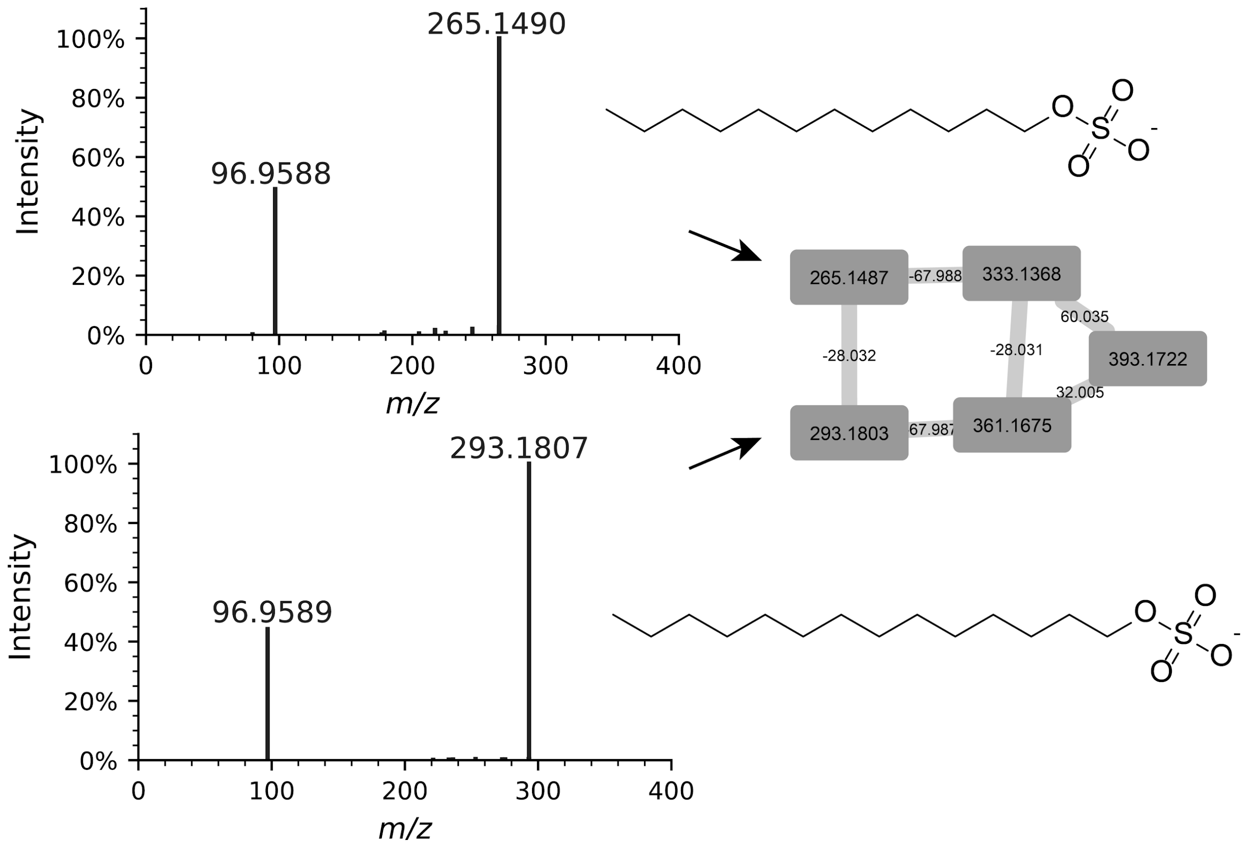

Figure S8. Proposed Annotations Observed in a Unannotated Component of the Negative Mode Molecular Network. The proposed annotation of dodecyl sulfate in the negative mode molecular network, node with an *m/z* 265.1487, and tetradecyl sulfate, node with an *m/z* 293.1803.
